# Use of H19 Gene Regulatory Sequences in DNA-Based Therapy for Pancreatic Cancer

**DOI:** 10.1155/2010/178174

**Published:** 2010-10-28

**Authors:** V. Scaiewicz, V. Sorin, Y. Fellig, T. Birman, A. Mizrahi, J. Galula, R. Abu-lail, T. Shneider, P. Ohana, L. Buscail, A. Hochberg, A. Czerniak

**Affiliations:** ^1^Department of Biological Chemistry, Institute of Life Sciences, The Hebrew University of Jerusalem, Edmond Safra Campus, Givat Ram, Jerusalem 91904, Israel; ^2^Department of Pathology, Hadassah University Hospital, Jerusalem 91120, Israel; ^3^Institut National de la Santé et de la Recherche Médicale U531, Institut Louis Bugnard, Institut Federatif de Recherche-31, Centre Hospitalier Universitaire Rangueil, 31403 Toulouse, France; ^4^Department of Surgery “A”, l Sheba Medical Center, Tel Hashomer 52621, Israel

## Abstract

Pancreatic cancer is the eighth most common cause of death from cancer in the world, for which palliative treatments are not effective and frequently accompanied by severe side effects. We propose a DNA-based therapy for pancreatic cancer using a nonviral vector, expressing the diphtheria toxin A chain under the control of the H19 gene regulatory sequences. The H19 gene is an oncofetal RNA expressed during embryo development and in several types of cancer. We tested the expression of H19 gene in patients, and found that 65% of human pancreatic tumors analyzed showed moderated to strong expression of the gene. In vitro experiments showed that the vector was effective in reducing Luciferase protein activity on pancreatic carcinoma cell lines. In vivo experiment results revealed tumor growth arrest in different animal models for pancreatic cancer. Differences in tumor size between control and treated groups reached a 75% in the heterotopic model (*P* = .037) and 50% in the orthotopic model (*P* = .007). In addition, no visible metastases were found in the treated group of the orthotopic model. These results indicate that the treatment with the vector DTA-H19 might be a viable new therapeutic option for patients with unresectable pancreatic cancer.

## 1. Introduction

Pancreatic cancer is the eighth most common cause of death from cancer in the world, with a very poor prognosis and a 5-year survival rate of about 3% [1, 2]. Due to late diagnosis because of the lack of marked symptoms, less than 20% of the patients are candidates for surgical resection—the only known possible treatment to cure this disease. Most of the patients present unresectable tumors and, because of the natural aggressiveness of the disease, micro- and macro-metastases are frequent in patients. Several clinical trials are being conducted combining different chemotherapies and radiotherapies, but none has yet shown more than a 10-month survival result [3, 4]. Given those facts, the need for new and more effective therapies is essential. 

Our group previously reported the use of DNA-based therapy for cancer treatment which drives the expression of diphtheria toxin A chain by the regulatory sequence of the H19 gene only into cancer cells [5, 6]. The human H19 gene is mapped at the short arm of chromosome 11, p15.5, homologous to a region of murine chromosome 7 [7]. It is an imprinted gene, expressed from the maternal allele in several tissues during embryo development and repressed right after birth [8]. H19 gene was shown to be re-expressed in several cancer tissues derived from those tissues which expressed the gene during embryonic development [9], such as bladder cancer [10], hepatocellular carcinoma [11], adrenocortical tumors [12], choriocarcinoma [13], colorectal cancer [14], ovarian carcinoma, and lung carcinoma [15]. H19 RNA shares all known characteristics of messenger RNA but has no known protein product [16]. H19 gene was also shown to play an important role in tumor growth; its expression caused differential expression of other genes [17]. It was demonstrated that oncogenes, tumor suppressor genes, and angiogenesis factors like TNF-*α* and NF*κ*B are regulated by the H19 gene [17]. 

The present work tested the feasibility of using the regulatory sequences of the H19 gene to express the diphtheria toxin A subunit for a DNA-based treatment for pancreatic cancer.

## 2. Materials and Methods

### 2.1. Gene Expression

#### 2.1.1. In Situ Hybridization

Tissue samples were prepared as described previously [13]. ISH was performed as described by Ariel et al. [11], using the digoxigenin labeled H19 RNA probe or by a modification of this method using H19-LNA (locked nucleic acid) Dig-labeled probe synthesized at Exiqon (See Table SA in Supplementary Mateial available online at doi:10.1155/2007/69141).

#### 2.1.2. RT-PCR Analyses

Total RNA was extracted from cell lines or frozen tissue, using TRI Reagent Solution, following manufacture's instructions (Sigma-Aldrich Israel Ltd., Rehovot, Israel). cDNA was synthesized starting from 2 *μ*g total RNA using the M-MLV Reverse Transcriptase kit following manufacturerr's instructions (Promega, Madison WI, USA). PCR reactions were carried out using GoTaq Green Master Mix (Promega), starting from 2 *μ*l cDNA in a final volume of 25 *μ*l, according to the manufacture instructions, in the presence of 0.1 *μ*g/*μ*l of each of the forward and the reverse primers. Primers designation sequences, and PCR conditions are detailed in Supplement Table A. The integrity of the cDNA was assayed by PCR analysis of the ubiquitous beta actin, which was used as internal control. As negative control for all PCR analysis, the reaction was carried out using water in the absence of cDNA.

### 2.2. Sequencing

For analysis of hamster H19 gene expression, RT-PCR was performed using 2 different sets of primers designed for mouse H19 gene. The resulting bands in the gel were purified using the Wizard SV Gel and PCR Clean-Up System kit, following the manufacture's instruction. The purified PCR product was sent to The Center for Genomic Technologies, The Hebrew University of Jerusalem. Sequences were verified by BLAST analysis using two different databases, Ensembl (http://www.ensembl.org/) and NCBI (http://www.ncbi.nlm.nih.gov/), and by sequence alignment using MAFFT version 6 (http://align.bmr.kyushu-u.ac.jp/mafft/online/server).

### 2.3. Cell Culture

The different cell lines used in this investigation were chosen because of their differences in the level of H19 gene expression and their response to transfection procedure. Human pancreatic adenocarcinoma cell lines CRL-1687, CRL-2119, CRL-1997, and CRL-2547 and ductal adenocarcinoma cell line CRL-1469 were obtained from the American Type Culture Collection (ATCC; Rockville, MD, USA) and cultured in 90% DMEM-F12 medium and 10% Fetal Bovine Serum (FBS). The hamster pancreatic ductal carcinoma cell line PC.1-0 was kindly provided by Dr. Buscail L. (Institut National de la Santé et de la Recherche Médicale U531, Institut Louis Bugnard, Institut Federatif de Recherche-31, Centre Hospitalier Universitaire Rangueil, 31403 Toulouse, France) and was cultured in 90% RPMI-1640 medium supplemented with 10% FBS. Antibiotic solution (penicillin (180 units/ml), streptomycin (100 *μ*g/ml), and amphotericin-B (0.2 *μ*g/ml)) were added to all medium solution. 

### 2.4. Plasmids and In Vitro Experiments

The reporter plasmids and the DTA-H19 construct were prepared as previously described [5, 6]. A total of 7–9 × 10^5^ cells/well were grown overnight in a twelve-well Nunc multidish. Cells were cotransfected with 2 *μ*g of the Luc-SV40 reporter vector and increasing concentrations of the DTA-H19 vector using the transfectant jetPEI (Polyplus, Illkirsh, France) according to manufacturer's instructions (N/P = 5). The luciferase activity in the cotransfected cells (DTA-H19 and Luc-SV40 vectors) was compared to that obtained in the cells transfected with Luc-SV40 only, in order to determine the cytotoxic effect of the DTA-H19 plasmid.

### 2.5. In Vivo DNA-Based Therapy

#### 2.5.1. Heterotopic Model for Pancreatic Cancer

Confluent cells were trypsinized and resuspended in PBS X1. A final volume of 100 *μ*l of cell suspension was injected subcutaneously into athymic nude mice (5–6 weeks old and 20–30 grs) purchased from Harlan (Zeist, The Netherlands). After tumor reached an approximated size of 3 mm × 3 mm, 3 treatments were given, with a 2-day interval between each treatment, by direct injection into the tumor. Treatment consisted of 25 *μ*g plasmid (DTA-H19 for treated group and Luc-H19 for control group) mixed with the nonviral transfectant polyethylenimine (Polyplus) (N/P = 6) diluted in 50 *μ*l of 5% w/v glucose (prepared according to manufacturer's instructions). Animals were sacrificed 3 days after the third treatment; tumors were excised for *ex vivo *measurements and for pathology and molecular studies.

#### 2.5.2. Orthotopic Model for Pancreatic Cancer

The following pancreatic cancer animal model was based on previous studies [18, 19]. 5–6-week-old male Syrian golden hamsters (70–80 grs) were purchased from Harlan and used to generate the orthotopic model. After anesthetization by subcutaneous injection of Ketamine and Xylazine (85 mg/kg and 15 mg/kg, resp.), the pancreas was surgically exposed, and 25 *μ*l of cell suspension containing 250,000 PC.1-0 cells in PBS X1 were injected subcapsularly into the pancreas using a catheter with a 27-gauge needle. Tumors reached a treatable size on day 7, and metastases were visible on day 9 following cell injection. 

After tumors were generated, the animals bearing the pancreatic tumors were treated at days seven and nine following cell injection. On day eleven, animals were sacrificed. The treatments were given under anesthesia, consisting of an intratumoral injection of 50 *μ*g of DNA vector (Luc-H19 for the control group and DTA-H19 for the treated group) resuspended in 25 *μ*l of saline solution. Before each treatment and before sacrifice, tumor sizes were measured using a caliber, and abdominal cavity was searched for visible metastases. Tumors were excised and their *ex vivo* dimensions were recorded following sacrifice and tumor measurement *in vivo*. The tumors were cut in the middle; one half of each tumor was fixed in formalin, processed, and embedded in paraffin for pathology studies, and the other half was frozen in liquid nitrogen for further molecular analysis. All experiments were performed according to the rules of the Animal Ethics Committee.

## 3. Results

### 3.1. H19 Expression in Human Pancreatic Cancer

**Figure 1:**
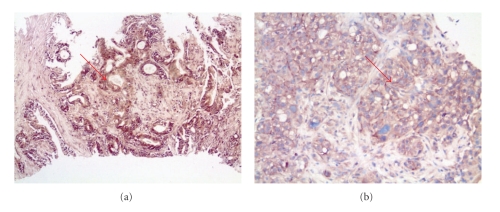
ISH of well-differentiated pancreatic carcinoma from two different patients. (a) Detection of H19 transcript using Dig-labeled H19 LNA-DNA probe by ISH. The arrow points to the positive hybridization signals within the cytoplasm of the cancer cells (10X magnification). (b) Detection of H19 transcript using Dig-labeled H19 LNA-DNA probe by ISH. The arrow points to the positive hybridization signals within the cytoplasm of the cancer cells (20X magnification). Tissue was later stained using Hematoxylin and Eosine.

Human pancreatic tumor samples from 49 patients were examined for H19 expression by ISH. Results showed that 65% of the tested samples were positive for H19 expression (Figures 1(a) and 1(b)).

### 3.2. H19 Expression in Cell Lines

**Figure 2:**
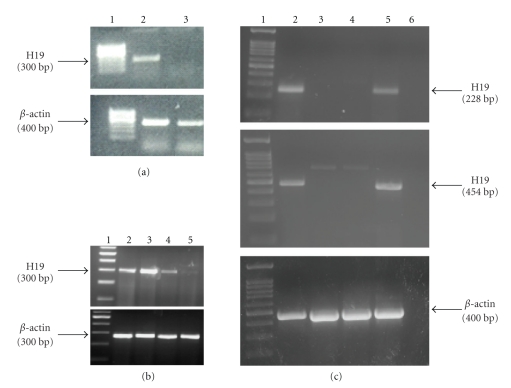
H19 RNA level in human and hamster pancreatic carcinoma cell lines. (a) Lane 1: 100 bp marker, Lane 2: CRL-1469 cells, Lane 3: PC.1-0 hamster cells. (b) Lane 1: 100 bp marker, Lanes 2–5: CRL-2547, CRL-1687, CRL-2119, and CRL-1997 human pancreatic carcinoma cells, respectively. (c) Lane 1: 100 bp marker; Lane 2: mouse control; Lane 3: PC.1-0 cells cultured under normoxia conditions; Lane 4: PC.1-0 cells cultured under hypoxia condition for 4 hours; Lane 5: tumors generated by PC.1-0 cells injection into nude mice back; Lane 6: negative control, no cDNA is present in the reaction mixture. The upper and the middle panels show the 228 bp and the 454 bp PCR products, respectively, obtained using two different primers (H19-mouse 228 and H19-mouse 454 as described in Supplementary data, Table A). The lower panel indicates the 400 bp *β*-actin internal control.

Three of the analyzed cell lines (CRL-2547, CRL-1687, and CRL-1469) showed high level of H19 expression (Figure 2(b), lanes 2 and 3 and Figure 2(a), lane 2, resp.), whereas the other two cell lines (CRL-2119 and CRL-1997) showed low levels of H19 expression (Figure 2(b), lanes 4 and 5, resp.). H19 expression was also analyzed on PC.1-0 cells, a hamster pancreatic carcinoma cell line. Since there is no report of the hamster's H19 gene sequence at the gene databases Ensembl and NCBI, primers designed originally for human and murine known H19 sequence were tested to determine H19 RNA level in hamster PC.1-0 cells. A faint band was visualized when primers designed originally for mouse H19 gene sequence were used (Figure 2(c), middle panel, lane 3). Previous studies showed that H19 gene expression may be induced by hypoxia and/or serum starvation conditions and that the H19 gene may play a role in tumorogenesis [17, 20]. Therefore, the level of H19 RNA in PC.1-0 was determined by RT-PCR extracted from cells cultured under hypoxia conditions for four hours, and also in total RNA extracted from tumors generated in athymic nude mice back by injecting 2*10^6^ PC.1-0 cells (cells injected were cultured under normal conditions). When amplifying total RNA from cells cultured under hypoxia, a weak band was visualized using the set of primers H19_mouse (Figure 2(c), lane 4, middle panel; primers 454 bp Supplement Table A). On the other hand, when RNA extracted from the PC.1-0 developed tumors was analyzed, strong bands were visualized using the same set of primers (Figure 2(c), lane 5, upper and middle panel). These strong bands were purified and sequenced. BLAST analysis and sequences alignment showed high homology between the PCR products and the available sequences of mouse and human on gene databases (data not shown). Despite the high homology between the sequences, further analysis must be performed in order to assure that the sequence corresponds indeed to the hamster H19 RNA.

### 3.3. DTA-H19 Vector Efficacy: In Vitro Studies

**Figure 3:**
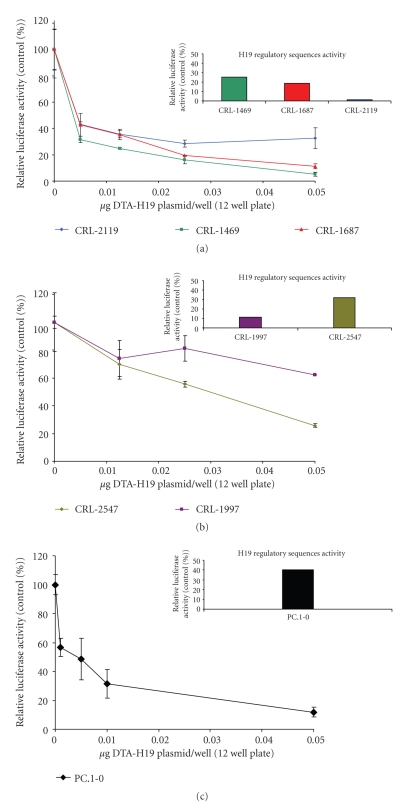
Reduction of Luciferase activity in human and hamster pancreatic carcinoma cell lines due to cotransfection with the DTA-H19 vector. The therapeutic potential of the DTA-H19 vector in different cell lines was measured as a reduction of Luciferase activity. (a) 90000 cells/well were cotransfected with 3 *μ*g of Luc-SV40 plasmid and the indicated amounts of DTA-H19. The luciferase assay was performed 48 hours after transfection. (c) 70000 cells/well were cotransfected with 3 *μ*g of Luc-SV40 plasmid and the indicated concentrations of DTA-H19. Transfection experiments were stopped after 24 hours, and reporter gene activity was assessed. The reduction in the Luc-SV40 activity in the cotransfected cells was compared to cells transfected with Luc-SV40 alone for each cell line. Bar graphs represent the relative Luciferase activity of the H19 promoter when cells were transfected with Luc-H19 plasmid.

In order to test the *in vitro* therapeutic potential of DTA-H19 plasmid, the human pancreatic carcinoma cells CRL-1687, CRL-2119, CRL-1997, CRL-2547, and CRL-1469 and hamster pancreatic ductal carcinoma cell line PC.1-0 were cotransfected with 2 *μ*g of Luc-SV40 at the indicated concentrations of DTA-H19 plasmids (Figure 3). Luciferase activity was measured 48 hours after transfection, and data was normalized to total protein per well. The toxic effect was calculated as the relative luciferase activity in the cotransfected cells compared to the activity in cells transfected only with the Luc-SV40 plasmid. The results showed that the DTA-H19 vector was effective in reducing the Luciferase activity in all the cell lines and that there is a dose-response effect (Figure 3). Exogenous H19 regulatory sequence activity was assayed by expressing the luciferase gene under the control of H19 regulatory sequences (Luc-H19 plasmid). Results showed that the cytotoxic effect of H19-DTA observed in the different cell lines is directly correlated to the luciferase activity expressed from the Luc-H19 plasmid (Figure 3).

### 3.4. DTA-H19 Treatment in Two Different Animal Models of Pancreatic Cancer

#### 3.4.1. Heterotopic Model

**Figure 4:**
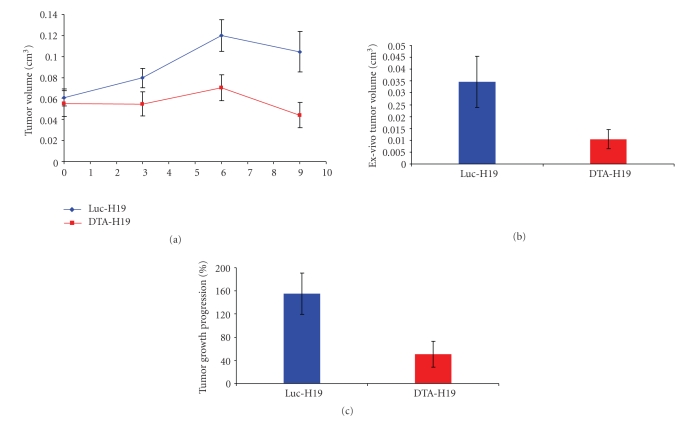
Heterotopic model for pancreatic cancer. (a) Average tumor volume of Luc-H19 group (*n* = 8) and DTA-H19 group (*n* = 7) during the experiment using CRL-1469 cells. Treatments were administrated on days 0, 3, and 6 by direct intratumoral injection after subcutaneous implantation of human pancreatic carcinoma cells in the back of nude mice. Three days after the last treatment, the animals were sacrificed. (b) Average of *ex vivo* tumor volume at the end of the experiment, tumors generated using CRL-1469 cells. (c) Tumor Growth Progression of Luc-H19 (*n* = 5) and DTA-H19 (*n* = 5) groups, in experiment using CRL-2547 cell line.

Tumors were generated into athymic nude mice back by subcutaneous injection of CRL-1469 cells and then treated with DTA-H19 (*n* = 7) or Luc-H19 (*n* = 8, control group) plasmid by direct injection into the tumor. Results showed that treatment with DTA-H19 resulted in an arrest of tumor growth, leading to a relative maintenance of tumor volume (Figure 4(a)). A significant difference in tumor growth progression between the two groups was found, indicating that the growth of the DTA-H19 treated group was arrested (*P*-value =  .036). This observation was confirmed by the final *ex vivo* tumor volume, which showed to be significantly smaller for the DTA-H19 group as compared to the control group (*P*-value =  .038) (Figure 4(b)). 

Similar results were obtained when the model was generated by injecting CRL-2547 cells (*P*-value <  .1) (Figure 4(c)).

#### 3.4.2. Othotopic Model

**Figure 5:**
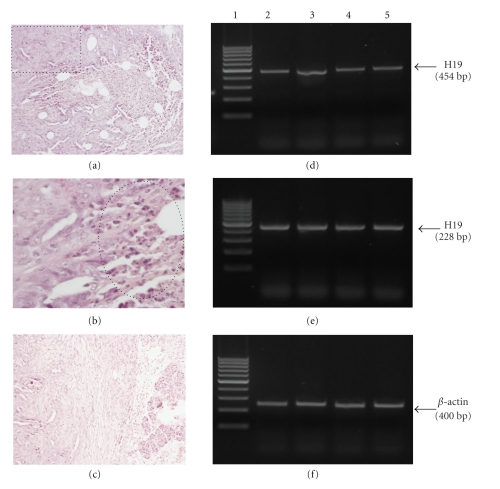
Histopathology analyses and H19 transcript detection in orthotopicaly induced tumors in the pancreas. (a) and (b) Both pictures show poorly differentiated carcinoma. The square in picture (a) delimits the malignant tissue; the circled area in picture (b) corresponds to necrotic interface (X20 original magnification for picture (a), X40 original magnification for picture (b). (c) Interface pancreatic acini (right), neoplasm (left), and desmoplastic reaction (middle) (X10 original magnification). Pictures were taken from paraffin sections and correspond to Giemsa stain. (d)–(f) Total RNA was extracted from frozen pancreatic tumors generated by intracapsular injection of PC.1-0 cells into hamsters pancreas. Lane 1: 100 bp marker, Lane 2: mouse control RNA, Lane 3: RNA extracted from tumor generated in the head of the pancreas, Lanes 4 and 5: RNA extracted from two different tumors generated in the pancreas tail in two different animals. (d) PCR products using primers H19_mouse 454. (e) PCR products using H19-mouse 228 primers. (f) Internal control *β*-actin mRNA.

A hamster model of pancreatic cancer was developed to evaluate the therapeutic efficacy of DTA-H19 for this indication. Seven days after cell implantation, all the animals presented visible and measurable tumors in the pancreas. The relevance of this orthotopic pancreatic carcinoma animal model was tested by studying the expression of the H19 gene which is a prerequisite to evaluate the therapeutic potential of the toxin expression vector in this animal model. All the analyzed samples from tumors generated in the pancreas showed high H19 expression when tested by RT-PCR (Figures 5(d) and 5(e)), signifying that our model was suitable for testing the DTA-H19 efficacy *in vivo*. Following H19 expression confirmation, histological studies were performed in order to determine if the tumors were compatible with pancreatic carcinoma. Histopathology analysis detected adenocarcinoma in the pancreas (Figures 5(a)–5(c)). All the tumors presented necrotic areas which were detected on the center of each tumor, caused by poor oxygen and nutrients supply (Figure 5(b)).

**Figure 6:**
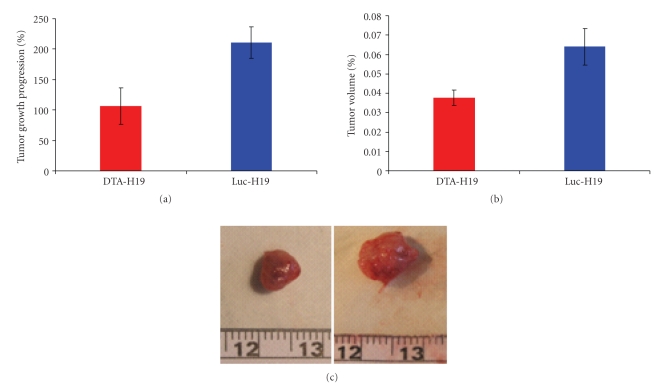
Effect of DTA-H19 treatment in Tumor Growth Progression and ex vivo tumor size on the orthotopic pancreatic carcinoma in the hamster. (a) Average of tumor growth progression of the DTA-H19 (*n* = 9) and Luc-H19 (*n* = 7) treated groups calculated as the ratio of tumor size at the end of the experiment (day 11 after cells implantation) compared to the size before treatment (day 7 after cells implantation). (b) Average of *ex vivo* tumor volume of the treated (DTA-H19) and control group (Luc-H19) measured after sacrifice (day 11). (c) The left picture corresponds to a tumor treated with DTA-H19 plasmid, and the right picture corresponds to a tumor from the control group.

Seven days after cell injection, 100% (*n* = 16) of the animals presented a single tumor in the pancreas. The average size of the tumor was 0.019 cm^3^, and some animals presented possible metastases. The animals were divided into 2 groups, the first was treated with 50 *μ*g of DTA-H19 plasmid and the second group with Luc-H19 plasmid (control group). The treatments were given at days 7 and 9 following tumor cells injection. As expected, DTA-H19 treatment was effective in inhibiting the tumor growth compared with the Luc-H19 treated tumors. While the control group showed a greater than 200% increment in tumor size, the DTA-H19 group showed approximately 100% (*P* <  .03) (Figure 6(a)). At the end of the experiment, the tumors were excised and their *ex vivo* size was determined. A significant difference on tumor size was detected when comparing DTA-H19 tumors to the control. The *ex vivo* tumor volume average of the DTA-H19 treated group was almost half that of the control group (*P*  <  .0075) (Figures 6(b) and 6(c)).

**Figure 7:**
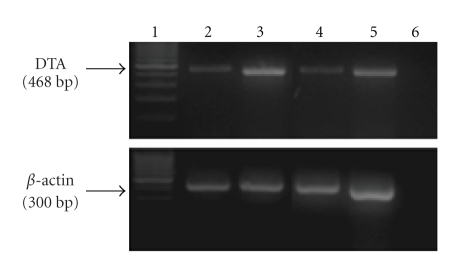
DTA expression in tumors samples. Total RNA was extracted from frozen pancreatic tumors treated with DTA-H19 plasmid. DNase-treated RNA and nontreated RNA samples were analyzed by RT-PCR for DT-A transcript level. Lane 1: 100 bp marker, Lanes 2 and 4: PCR products from DNase-treated RNA, Lanes 3 and 5: PCR products from nontreated RNA, Lane 6: negative control, no cDNA is present in the reaction mixture. The upper panel shows the 468 bp DT-A product, and the lower panel shows the *β*-actin internal control.

The level of DTA transcript was monitored by RT-PCR analyses (Figure 7). All the samples showed the presence of DTA transcript verifying that the intratumoral injection of the plasmid leads to transfection and expression of the DTA gene.

In order to test the systemic toxicity of DTA-H19, different organ samples were collected from animals treated with the vector at terminal sacrifice and subjected to histopathology examination. No gross or microscopic significant alterations were noted in the following organs: pancreas, liver, lung, bowel, spleen, heart, kidney, adrenal gland, lymph-node, salivary glands, gall-bladder, urinary-bladder, and brain (data not shown). 

Although treatment with naked DNA proved to be sufficient for inhibiting tumor growth progression, we tested if the use of a transfection enhancer reagent such as PEI could improve the efficacy results. According to the developed animal model described previously, PC.1-0 cells were injected into the pancreas. On day 7, when tumors were visible, they were treated with 100 *μ*l of a complex containing 50 *μ*g of DTA-H19 plasmid and *in vivo *PEI (prepared according to manufacturer's instructions). This treatment was repeated on day 9, and animals were sacrificed on day 11. The results obtained showed that no significant improvement in the therapeutic effect was found when PEI was used as a carrier for delivering the plasmid as compared to that obtained using naked DNA (data not shown).

As mentioned above, metastases occurrence was also analyzed during the present experiment. Metastases were visible mostly on day 9, after the first treatment, while few animals showed metastases on day 7. All the animals in the control group showed multiple visible metastases at the end of the experiment, whereas 70% of the animals treated with DTA-H19 did not show visible metastases, and the number of metastases present in those animals was significantly lower (Table 1).

## 4. Discussion

The present study proposes a successful approach for the differential killing of pancreatic cancer cells, without affecting the surrounding normal cells. This approach consists of a DNA-based therapy expressing a toxin under the control of regulatory sequences of a differentially expressed gene, the H19 gene. It was previously demonstrated that the DTA-H19 construct was able to kill tumor cells both *in vitro *and *in vivo *in animal models for bladder cancer and colorectal liver metastases [5, 6]. This toxin vector was used in order to kill pancreatic cancer cells either in culture or in tumors developed in heterotopic and orthotopic animal models of pancreatic cancer. We successfully demonstrated by ISH analysis that the H19 gene is expressed in 65% of the analyzed human pancreatic cancer samples (*n* = 49, Figure 1), indicating that treatment of human pancreatic cancer with the DTA-H19 is very likely to be effective in this disease.

H19 promoter activity was analyzed in all human and hamster cell lines using a plasmid carrying the luciferase gene under the control of H19 regulatory sequence. A relatively high activity of the regulatory sequences was detected in all the tested cell lines, and this activity was correlated with the cytotoxic effect observed when using these sequences for driving the expression of the diphtheria toxin A chain (Figure 3). The effect of the DTA-H19 plasmid cotransfected into cells with a second plasmid which encodes the luciferase gene under the control of the SV40 regulatory sequence caused inhibition of the luciferase activity at a dose-response manner in all the studied cell lines (Figure 3). The highest amount of the DTA-H19 plasmid used in cotransfection with Luc-SV40 construct was about 40 times lower than that of the Luc-SV40 plasmid. Therefore, the observed effect is more likely due to the cytotoxic effect of the DTA-H19 plasmid expression and not the result of competition between the two plasmids for the entry into the cell. These results lead us to test the toxic effect of the plasmid *in vivo*. 

We tested the cytotoxic effect of the therapeutic vector both in a heterotopic and an orthotopic model for pancreatic cancer which are complementary and allow the study of tumors with distinctive histopathologies. The results obtained in the heterotopic model using CRL-1469 cells showed a significant difference in TGP (*P*  =  .036) between the DTA-H19 treated group and the control group. These results were confirmed by the reduction in tumor size *ex vivo*, where the average tumor size of the DTA-H19 group was almost 1/3 the size of the control group (*P* =  .037) (Figure 4). Furthermore, when using a different cell line to generate the tumors, CRL-2547 cells, similar results were obtained, corroborating that the treatment with DTA-H19 was effective in arresting tumor growth.

We successfully developed an orthotopic animal model for pancreatic cancer. The fact that all the animals developed a similar size of tumors after PC.1-0 cell injection ensured its reproducibility. H19 expression was previously determined in the developed tumors by RT-PCR analyses in order to validate the use of this animal model in evaluating the potential therapeutic effect of the toxin vector. High levels of H19 gene expression were detected in the developed tumors even though no expression was detected in cultured cells (Figure 5), which is in agreement with previous observations establishing that H19 gene expression may be induced by hypoxia and/or serum starvation conditions developed following injection of tumor cells into the animal tissue [17]. Animals presenting pancreatic tumors were then treated with either DTA-H19 plasmid or the control plasmid (Luc-H19) administrated twice by direct injection into the tumor. We demonstrated that although the treatment consisted of an injection of naked DNA into the tumor, the vector indeed entered the cells and the H19 sequence was effective in leading to the DT-A gene expression (Figure 7). It was previously observed that no significant difference in efficacy was found when comparing the use of naked DNA to DNA-PEI when administrated by direct injection into the tumor [6].

The activity of the H19 promoter was demonstrated in the human and hamster pancreatic tumor cells tested, leading to DTA expression (Figures 2 and 3). Therefore, inhibition of the growth of DTA-H19 treated tumors in the hamster results from the action of the DTA produced (Figure 6(a)). Intratumoral injection of the toxin vector under the control of the H19 regulatory sequences induced a 50% decrease in the median tumor volume as compared to that of the tumor treated with the Luc-H19 vector (Figures 6(b) and 6(c)). Although the tumors were not completely ablated, the treatment with the toxin vector significantly reduced the growth rate of the DTA-H19 treated tumors as compared to the control group. We described the appearance of visible metastases on day 9 after cell injection. We observed that after DTA-H19 treatment, 67% of the treated animals did not present visible metastases. The number of metastases found in the rest of the treated group (33%) was significantly lower and more localized than those of the control group. These results indicate that the lack of visible metastases in 2/3 of the animals is related to the reduction of tumor progression in the DTA-H19 treated animals. Metastases developed in the toxin-treated group may have been already present before the first treatment, as micrometastases, and therefore their growth was not completely prevented by the intratumoral treatment. Successful treatment depends on the activity of the tumor-specific promoter. The H19 promoter is known to be differentially activated in various tumor types and to show none or undetectable activity in normal tissue [5, 6]. The presented results lead us to advocate a new DNA-based treatment for pancreatic cancer, inducing cytotoxic effect in cancer cells by intratumoral injection of a naked DNA vector containing the DT-A gene under the control of H19 regulatory sequences for human pancreatic cancer.

## Supplementary Material

Table SA. Probe and primers designed for H19 and DTA gene expression analysis.

## Figures and Tables

**Table 1:** Metastases observation developed in the hamster orthotopic pancreatic carcinoma model. Animals presenting pancreatic tumors were treated twice with DTA-H19 or Luc-H19 plasmids. Before each treatment, abdominal cavity was searched for metastases in both groups, the treated group DTA-H19 (*n* = 9) and the control group (*n* = 7). Most metastases appeared visible on day 9.

Group	Animals with metastasis on day 7/total	Animals with metastasis on day 9/total	Animals with metastasis on day 11/total
**DTA-H19**	**0/9**	**3/9**	**3/9 **Metastases in liver, stomach, or intestine (*n*≤ 2)
**Luc-H19 **(control group)	**1/7**	**4/7**	**7/7 **Multiple abdominal metastases (*n*> 3)
