# Supplementary material for: Use of H19 Gene Regulatory Sequences in DNA-Based Therapy for Pancreatic Cancer
Source: J Oncol. 2010 Oct 28;2010:178174. doi: 10.1155/2010/178174 (PMC2967839; doi:10.1155/2010/178174)
Supplement: Supplementary file 1 — Table SA. Probe and primers designed for H19 and DTA gene expression analysis. [file 178174.f1.pdf]

Supplementary Table A

Designation	Sequence	PCR Conditions
H19_Probe	5'-TCCTCTAGCTTCACCTTCCAGAG-3'	
H19_Human Forward<br><br>H19_Human Reverse	5'-CCGGCCTTCCTGAACA-3'<br><br>5'-TTCCGATGGTGTCTTTGATGT-3’	94°C for 5 min, followed by 30 cycles of 94°C for 30 sec, 59°C for 45 sec, 72°C for 1 min, final 72°C for 5 min.
H19_mouse228 Forward<br><br>H19_mouse228 Reverse	5’-CATGTCTGGGCCTTTGAA-3’<br><br>5’-TTGGCTCCAGGATGATGT-3’	94°C for 5 min, followed by 30 cycles of 94°C for 30 sec, 52°C for 45 sec, 72°C for 1 min, final 72°C for 5 min.
H19_mouse454 Forward<br><br>H19_mouse454 Reverse	5’-TCTCCAGCAGAGGTGGATGT-3’<br><br>5’-GCAGAGTTGGCCATGAAGAT-3’	94°C for 5 min, followed by 30 cycles of 94°C for 30 sec, 56°C for 45 sec, 72°C for 1 min, 72°C for 5 min.
DTA Forward<br><br>DTA Reverse	5'-TTCGTACCACGGGACTAAACCTGG-3'<br><br>5'-CCACGTTTTCCACGGGTTTCAA-3'	95°C for 5 min, followed by 30 cycles of 95°C for 1 min, 52°C for 40 sec, 72°C for 30 sec, final 72°C for 5 min.
